# Impact of Dental Treatment on Oral Health-Related Quality of Life of Patients

**DOI:** 10.7759/cureus.38625

**Published:** 2023-05-06

**Authors:** Rupali Malik, Thanveer K, Vikas Singh, Ankita Jain, Subhajit Mitra, Sweety Singh

**Affiliations:** 1 Public Health Dentistry, Teerthanker Mahaveer Dental College and Research Centre, Moradabad, IND; 2 Oral and Maxillofacial Surgery, Teerthanker Mahaveer Dental College and Research Centre, Moradabad, IND; 3 Pediatric and Preventive Dentistry, Teerthanker Mahaveer Dental College and Research Centre, Moradabad, IND

**Keywords:** functional limitation, ohip-14, non-invasive dental treatment, invasive dental treatment, dental health, oral health-related quality of life

## Abstract

Objective: It is crucial to understand how individuals perceive the impact of oral disorders and the treatment associated with or received for those disorders on their quality of life. A relatively new but quickly spreading concept of oral health-related quality of life (OHRQoL) that notably affects three fields, clinical dental practice, dental research, and dental education makes it feasible to figure out the relationship between oral health and its impact on the quality of life of an individual. OHRQoL can be measured in various ways; the most well-liked method uses a multiple-item questionnaire. There haven't been any prior attempts to compare the effects of various invasive and non-invasive dental therapies on OHRQoL, even though few studies have been conducted to evaluate the OHRQoL among patients undergoing independent dental procedures. Such a comparison would aid in our understanding of not only how various dental conditions affect OHRQoL, but also whether or not a patient’s OHRQoL has improved as a result of various therapies for these diseases.

Method: A longitudinal study was conducted on patients receiving invasive and non-invasive dental treatment at Teerthanker Mahaveer Dental College and Research Centre, Moradabad. A two-part questionnaire, the first part of which consists of questions related to the demographic details of the patient and the second part consisting of a set of 14 questions of the oral health impact profile (OHIP)-14 for assessing the OHRQoL, was used in the study. Patients' baseline OHRQoL was assessed before the commencement of any treatment by the interview method and follow-up OHRQoL was assessed three days, seven days, one month, and six months post-treatment telephonically. The OHIP-14 contains 14 items on the frequency of adverse impacts caused by oral conditions and the patients were asked to rate each item on a 5-point Likert scale as 0=never; 1=hardly ever; 2=occasionally; 3=fairly often; 4=very often.

Results: The results obtained after compiling and analyzing the data from a total sample of 400 indicate that the mean difference in the OHIP score at different time intervals between the groups who undertook invasive and non-invasive treatment was significant as the p-value was less than 0.05. In addition, it was observed that the mean difference at baseline was statistically significant in the invasive and non-invasive groups as the p-value is less than 0.05. At the domain level, the mean score at each domain was higher in the invasive group as compared to the non-invasive treatment group after three days and seven days of treatment. The mean difference between the group treated with invasive treatment on day three and the group treated with non-invasive treatment on day seven was statistically significant as the p-value is less than 0.05. The mean score was high in the invasive group as compared to the non-invasive group after one month and six months of treatment.

Conclusion: The present study was conducted to assess dental treatment’s impact on oral health-related quality of life in patients attending Teerthanker Mahaveer Dental College and Research Centre, Moradabad. Results from this study indicated that both types of treatments either invasive or non-invasive have significantly influenced the OHRQoL. Post-treatment OHRQoL improved at different intervals after receiving either treatment.

## Introduction

The public health community views health as a multifaceted concept, health has historically been measured narrowly and from a deficit viewpoint [1]. Given that medical and public health advancements have resulted in cures and better therapeutic options for existing diseases as well as deferred death rates, it was reasonable to assume that professionals who monitor health outcomes would begin to evaluate the health of a population in terms of both rescuing and enhancing the quality of those lives.

This wide and comprehensive idea of "quality of life" (QoL) frequently incorporates subjective assessments of both good and bad elements of life [2]. In the 1980s, the evolution of the concept of health-related quality of life (HRQoL) and its components took place to cover all facets of the general quality of life that have been shown to have an effect on health, be it physiological or psychological [3-6].

The general health of an individual is intimately associated with his or her oral health. Each and every condition that negatively affects oral health may have profound effects on an individual’s physiological, mental, and social health [7]. Oral health and the oral cavity should be considered as a part of a complete body, any hindrance to which can have an ill effect on overall health and life’s quality.

The majority of oral health issues lead to a high rate of dissatisfaction in terms of esthetics, appearance, workability, and their day-to-day normal routine like eating, sleeping, talking, etc. among the various age groups in the population. A multidimensional construct called OHRQoL determines a person's satisfaction with their dental health as well as their self-esteem and comfort during eating, sleeping, and socializing. OHRQoL, despite being a new concept during the past few decades, has significant implications for dental research and clinical practice. It is the outcome of interactions among factors affecting oral health, social and environmental factors, and other systems in the body [8]. It has extensive uses in surveys and medical research. OHRQoL is crucial to overall health and well-being. In fact, the WHO acknowledges it to be a crucial component of the Global Oral Health Program. Acknowledging the importance of life’s quality that is associated with oral health, researchers have developed various approaches to measure OHRQoL out of which the most frequent and accessible one is the multiple-item questionnaire oral health impact profile (OHIP-14) proposed by Slade and Spencer based on the model proposed by Locker [6]. This is an instrument that is capable of edging towards social and psychological aspects by means of self-perception and assessment of the effects caused in quality of life. OHIP-14 is a scale that not only provides insight into the level of discomfort, disability, and/or dysfunction people feel as a result of their oral condition but also helps to determine an individual’s perception of the social impact of the oral disorder on their wellbeing.

Few studies have been done to assess the OHRQoL among patients receiving stand-alone dental treatments like oral surgery or orthodontics, however, a comparison of the impact of various invasive dental treatments and non-invasive dental treatment on OHRQoL has not been attempted before [9]. Such a comparison would help us better understand not only the impact of different dental diseases on the OHRQoL but also will throw light on the betterment of the OHRQoL of patients receiving various treatments. From a dental public health perspective this study will help us in understanding OHRQoL as a valid indicator of unmet needs and intervention outcomes.

## Materials and methods

A longitudinal study was conducted on a total of 400 patients receiving invasive and non-invasive dental treatment in Teerthanker Mahaveer Dental College and Research Centre, Moradabad, to assess the impact of dental treatment on OHRQoL, by using a two-part questionnaire with content validity index of 1 and Cronbach alpha value of 0.8, whose first section comprises inquiries about demographic information and the second part comprises a set of 14 questions of the OHIP-14 for assessing the OHRQoL. Here it is mentioned as invasive and non-invasive procedures and hence individual procedure QOL is not mentioned in the study results. OHIP-14 consists of seven components or domains, each component/domain consists of two items thus collectively making it OHIP-14. Domain 1 is named as functional limitation the two items of which are trouble pronouncing some words and taste had worsened. Domain 2 is named physical pain the two items of which are painful aches and uncomfortable eating food. Domain 3 is named as psychological discomfort the two items of which are self-conscious and tense feeling. Domain 4 is named as physical disability the two items of which are unsatisfactory diet and interruption of meals. Domain 5 is named as psychological disability the two items of which are difficult to relax and feeling embarrassed. Domain 6 is named as social disability of which the two items are being a bit irritable and difficulty doing usual jobs. Domain 7 is named handicap of which the two items are life less satisfying and totally unable to function.

Inclusion criteria

All the patients aged 18 years and older attending Teerthanker Mahaveer Dental College and Research Centre were included in the study. Patients with any debilitating systemic disorder, those receiving orthodontic treatment as it was a long-term treatment, those with communication difficulties, and those who refused to provide informed consent were excluded from the study. 

Data collection

A two-part questionnaire was used in the study, the first part of which consisted of questions related to certain demographic details of the patient and the second part consisted of a set of 14 questions of the OHIP-14 for assessing the OHRQoL. A validated Hindi version of the OHIP-14 questionnaire was used in this study. The adapted Hindi questionnaire was used in a pilot study with 30 patients in our area. The pilot study's Cronbach alpha value was 0.89, showing good internal consistency. In order to gather the primary study data, the research team used this common and reliable Hindi questionnaire. It took between 5 and 12 minutes to complete the questionnaire, which was found to be straightforward and simple to understand.

Patients’ baseline OHRQoL was assessed prior to the commencement of any treatment by the interview method and follow-up OHRQoL was assessed three days, seven days, one month, and six months post-treatment telephonically. The OHIP-14 contains 14 items on the frequency of adverse impacts caused by oral conditions and the patients will be asked to rate each item on a 5-point Likert scale as 0=never; 1=hardly ever; 2=occasionally; 3=fairly often; 4=very often.

Statistical analysis

The data collected were analyzed by applying descriptive and inferential statistical analysis using SPSS version 23.0 (Chicago, IL: IBM Corp.). Repeated measures ANOVA was carried out to analyze the difference within the respondent’s OHIP scores at baseline, after three days, after seven days, after one month, and after six months. Mauchly’s test of sphericity was carried out to assess the OHIP scores among the respondents. The Bonferroni post hoc test was used to assess the mean difference among the respondents at different time intervals. The intergroup comparison for the mean scores and frequencies between two independent groups was done using an unpaired/independent t-test. The significance level for results was fixed at a value of 5%.

## Results

**Figure 1:**
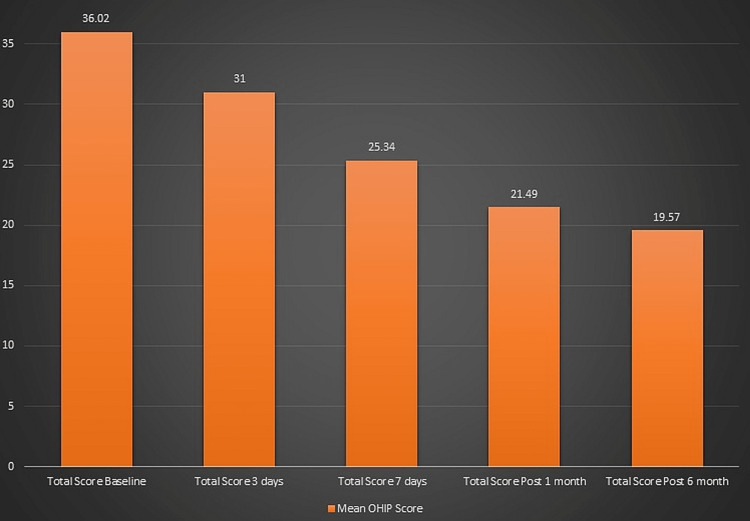
Descriptive statistics of OHIP scores of subjects. OHIP: oral health impact profile

The study sample consists of 252 (63%) male and 148 (37%) female patients. One hundred sixty-two subjects (40.5%) were from rural areas and 238 subjects (59.5%) were from urban zones. It was observed that there was a difference in both the minimum and maximum score of OHIP recorded at baseline compared to that after three days of treatment, after seven days of treatment, after one month of treatment, and after six months of treatment for all the subjects. The mean OHIP score at baseline was 36.02, after three days it was 31.00, after seven days the OHIP score was 25.34. The mean OHIP score reduced to 21.49 after one month and further to 19.57 after six months (Figure 1).

**Figure 2:**
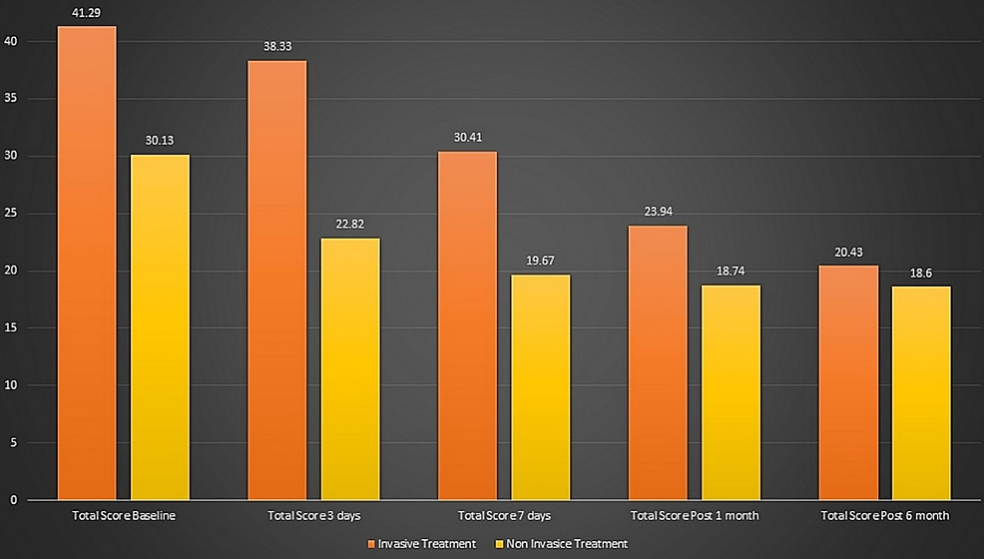
Mean OHIP score with respect to the treatment undertaken. OHIP: oral health impact profile

Out of the 400 subjects included, 211 subjects received invasive treatment and 189 received the non-invasive type of treatment. The mean OHIP score at baseline was 41.29 and 30.13 in the subjects who had invasive and non-invasive treatment, respectively. For the subjects who undertook invasive treatment, the mean score was 38.33 after three days, 30.41 after seven days, 23.94 after one month, and 20.43 after six months. The subjects who undertook non-invasive treatment had a mean OHIP score of 22.82 after three days, 19.67 after seven days, 18.74 after one month, and 18.6 after six months (Figure 2).

**Figure 3:**
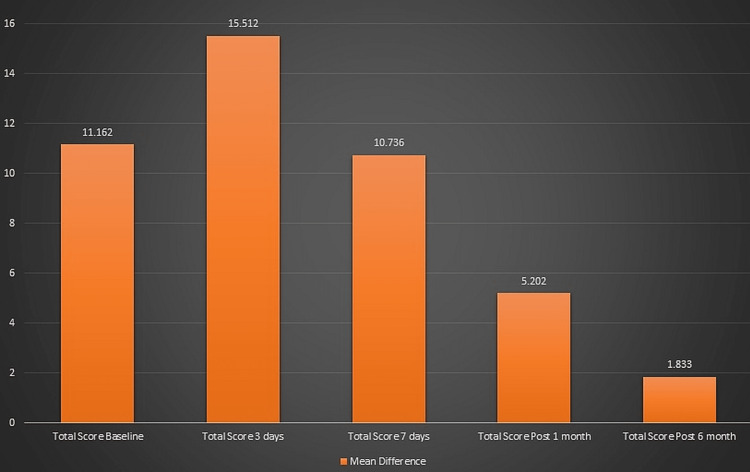
Mean difference of the OHIP scores between invasive treatment and non-invasive treatment groups. OHIP: oral health impact profile

Patients who underwent invasive treatment were found to have domain 2 which is associated with physical pain as the most affected domain at baseline and the least affected domain was domain 1 or functional limitation and domain 6 which is associated with social disability. After three days, seven days, one month, and six months of receiving invasive treatment, a reduction was seen in the mean score in all the domains, except in domain 1 and domain 7. Patients who received the non-invasive type of treatment were found to have domain 2 as the most affected domain at baseline and the least affected domain was domain 5 and domain 6. After three days, seven days, one month, and six months of receiving non-invasive treatment, a reduction was seen in the mean score in all the domains. The mean difference in OHIP between each of the time intervals was found to be statistically significant (p<0.05) (Figure 3).

Results of the independent samples t-test show that the mean difference in the OHIP score at different time intervals between the groups who undertook invasive and non-invasive treatment was significant as the p-value was less than 0.05. The mean score at each domain was higher in the invasive group as compared to the non-invasive treatment group after three days and seven days of treatment and was statistically significant (p<0.05). The mean difference after one month between the groups who got invasive treatment was statistically significant as compared to the group who received non-invasive treatment (p<0.05) except in domain 6 (p>0.05). The mean score between the group who got invasive treatment was statistically significant as compared to the group who received non-invasive treatment except in domains 3 and 6. The mean score was high in the invasive group as compared to the non-invasive group except in domain 5 where the mean score in the non-invasive treatment was more than in the invasive treatment group with respect to domain 5 (Table 1).

**Table 1:** Result of independent samples t-test to assess the mean difference in the OHIP score at different time intervals between the groups who undertook invasive and non-invasive treatments. df: degree of freedom; OHIP: oral health impact profile

	t	df	p-Value	Mean difference	95% confidence interval of the difference	
					Lower	Upper
Baseline	17.008	398	0.000	11.162	9.872	12.452
3 days	21.799	398	0.000	15.512	14.113	16.911
7 days	13.308	398	0.000	10.736	9.150	12.322
1 month	10.504	398	0.000	5.202	4.229	6.176
6 months	5.945	398	0.000	1.833	1.227	2.440

## Discussion

Results from the present study showed that both types of treatments either invasive or non-invasive have significantly influenced the OHRQoL but an invasive type of treatment influenced the OHRQoL more significantly than a non-invasive type of treatment. After receiving the treatment, the mean score had reduced in both invasive and non-invasive groups when examined on the third day, seventh day, after one month, and after six months of the treatment. Similar results were observed in a study where the use of an invasive mode of treatment was done and the OHRQoL was improved [10]. Nickenig et al. assessed the impact of dental implants on OHRQoL and found that the maximum score on OHIP-G 21 scale was before the treatment which was reduced to an extent in the healing period and reduced significantly by the completion of the treatment thus improving the OHRQoL [11].

Eitner et al. undertook a study to see how patients' and doctors' perceptions of their quality of life (OHRQoL) would alter prior to, during, and following prosthodontic implant therapy [12]. The median OHIP score was found to be reduced after the treatment in both scenarios which is similar to our study. Similar results were also observed for the non-invasive type of treatment in a study conducted by Wahbi and Elamin [13]. A study by Nikolovska and Petrovski also presented a statistically significant difference in the total OHIP score prior to and after a prosthodontic treatment with full dentures [14].

In our study, OHRQoL of patients after receiving the treatment significantly improved at different intervals. The same was also seen in the results of a study conducted by Jowett et al. [15]. In patients who had received invasive treatment, the most affected domain at baseline was 2 which is associated with physical pain. In patients who had received the non-invasive type of treatment the most affected domain at baseline was physical pain. Social disability scored least in both invasive and non-invasive treatments. Similar results were found in a study conducted by Nikolovska and Petrovski [14]. Shaghaghian et al. in their study revealed that physical disability and physical pain were the most problematic aspects [16]. Results from a study by Eitner et al. showed that the psychological dissatisfaction scale of the OHIP represented the most important factor for the patients who received dental implants [12].

Invasive treatment resulted in a significant decrease in the mean score across all domains from baseline to day three, with the exception of functional limitation and handicap, suggesting that patients still had trouble speaking clearly or that their sense of taste had gotten worse and that they felt their lives were less satisfying overall or were completely unable to function.

McGrath et al. observed similar deterioration in QoL in the immediate post-operative period following third molar surgery [17]. In a study by Eitner et al., in their assessment of OHRQoL for patients treated with dental implants at different time intervals they observed that the maximum OHIP score was found at the healing phase which is the intermediate phase when compared to pre-operative and post-operative phases [12]. White et al. reported that after the third molar surgery, it took an average of three days to get back to daily activities and social life and five to seven days to get over the inability to chew properly and get back on a regular diet [18]. Chou et al. found that there was a significant (moderate) difference between pre- and post-surgery physical pain (psychological discomfort) scores among their study participants [19]. Saito et al. reported that there was a significant difference between the QoL of periodontal surgery patients before and after treatment [20].

After receiving a non-invasive type of treatment there was a significant reduction in the mean score of each domain from baseline to the third day, seventh day, after one month, and after six months of receiving the treatment thus showing improvement in OHRQoL. Similar results were found in a study conducted by Goel and Baral where the effect of non-surgical periodontal therapy on OHRQoL was studied using the OHIP-14 score [21]. The outcomes were statistically significant and demonstrated that the OHRQoL of people with periodontal disease who received therapy improved. Our study shows no statistical significance among the mean OHIP score of male and female respondents or among the respondents who live in rural or urban areas.

Considering the methodology of our study, there are some limitations that should be noted. Due to convenience sampling and the fact that only patients who consented to participate were included, it is possible that those with less or more advanced stages of the disease weren't included.

## Conclusions

This study was conducted to assess the impact of dental treatment on oral health-related quality of life of patients, and the results showed that both types of treatments either invasive or non-invasive significantly influenced the OHRQoL. After invasive treatment, there was a slight deterioration in OHRQoL on third day of the invasive treatment. An improvement in OHRQoL was observed on the seventh day, after one month, and after six months of the invasive treatment. After receiving a non-invasive type of treatment there was a significant reduction in the mean score of each domain from baseline to the third day, seventh day, after one month, and after six months of receiving the treatment thus showing improvement in OHRQoL. A standardized OHRQoL measure should be part of all oral health assessments to provide a profile of the effects of oral disorders on people's everyday lives. OHRQoL assessments can be used to create strategic goals for improvements in oral health globally and are crucial in the advocacy of oral health policy.
